# Growing Old Too Fast: A Rare Case of Werner Syndrome

**DOI:** 10.7759/cureus.4743

**Published:** 2019-05-24

**Authors:** Anahat Kaur, Punita Grover, Anas Albawaliz, Mahak Chauhan, Brandon Barthel

**Affiliations:** 1 Internal Medicine, University of Missouri-Kansas City School of Medicine, Kansas City, USA; 2 Hematology and Oncology, University of Cincinnati, Cincinnati, USA; 3 Endocrinology, University of Missouri-Kansas City School of Medicine, Kansas City, USA

**Keywords:** werner syndrome, progeria

## Abstract

Werner syndrome (WS) is rare adult-onset progeria characterized by premature aging and early death. Patients develop normally until adolescence and usually present in early adulthood. Our case highlights a common presentation of this uncommon disease, wherein a 29-year-old non-obese male with no known risk factors developed uncontrolled diabetes, hypertriglyceridemia, and rapidly progressive atherosclerotic vascular disease. Careful observation with attention to the presence of characteristic physical features and subsequent genetic testing helped diagnose the patient with this uncommon progeroid syndrome. Our case adds to the literature about this rare disease especially in patients of middle-eastern descent and also highlights the importance of having a high index of suspicion for WS when the initial clinical presentation is atypical.

## Introduction

Werner syndrome (WS) is rare adult-onset progeria characterized by premature aging and early death. Patients develop normally till adolescence and usually present in early adulthood [1]. Our case highlights a common presentation of this uncommon disease.

## Case presentation

A 29-year-old Iraqi gentleman presented to the clinic for the management of diabetes mellitus (DM). He had been given a diagnosis of type 1 DM at the age of 19. He did not have any medical care for the past two years and had not been on insulin. Past history was significant for hypothyroidism and bilateral cataract surgery at the age of 19. Examination showed a thinly-built male with the body mass index of 19. He had gray hair, beak-like nose, and thin limbs with little subcutaneous fat. 

Work-up for diabetes revealed an elevated C-peptide and negative beta-cell autoantibodies, characteristic of type 2 DM. He was started on a typical insulin regimen of 1Unit/kg/day but needed to be quickly escalated to 2 units/kg/day, indicating severe insulin resistance. He had severe hypertriglyceridemia (triglyceride level of 3900 mg/dl) which needed three lipid-lowering agents. Over the course of the next few months, he was found to have three-vessel coronary artery disease requiring bypass surgery. He also developed severe right lower extremity ischemia due to extensive atherosclerosis and underwent multiple angioplasties. Despite appropriate medical and interventional management, his peripheral vascular disease worsened and he eventually required right leg amputation. Workup for an underlying hypercoagulable disorder was unrevealing. Additionally, his aged appearance, lipodystrophy, and the presence of diseases typical for an older population raised the suspicion of Werner syndrome. Whole genome sequencing was done and the patient was found to be homozygous for WRN mutation, confirming the diagnosis of Werner syndrome. The patient eventually left the country and was lost to follow-up.

## Discussion

WS is an autosomal recessive disorder [2]. It was first described by a German medical student, Otto Werner, in 1904 who reported a family of four siblings with cataracts and scleroderma [3]. Patients usually present in the second and third decades of life with characteristic clinical features of skin atrophy, ‘pinched’ faces, gray and thin hair, hoarse voice, lipodystrophy, bilateral cataracts, diabetes, atherosclerosis, skin ulcers, hypogonadism, and osteoporosis. Accurate diagnosis of WS is critical for prognosis since these patients are at increased risk of developing malignancies and have an average life expectancy of around 54 years [4]. Neoplasms and myocardial infarction are common causes of death in WS [1].

Of note, WS has been reported in several populations, with significantly high prevalence in Japan (estimated frequency of 1:100,000) [5]. The incidence of WS in the United States is 1 in 200,000 [6]. Other ethnicities in which this syndrome has been reported include Sardinian, Indian, Pakistani, Moroccan, Turkish, and Dutch patients [5,7].To the best of our knowledge, there is only one case report mentioning an Iranian family with four siblings showing signs of WS [8]. No other WS cases have been reported in patients of middle-eastern origin (Iraq, in our case). This makes our case an addition to the registry of ethnicities in which this disease has been reported.

Diagnostic criteria for WS as suggested by Takemoto et al. are listed in Table 1 [9]. For a confirmed diagnosis of WS, all cardinal signs need to be present or gene mutation should be present in addition to three or more cardinal signs. WS is suspected when either two or more cardinal signs are present or one to two cardinal signs are present in addition to other signs [9].

**Table 1:** Diagnostic criteria for Werner syndrome

Cardinal signs and symptoms (onset between 10 and 40 years of age)
1. Progeroid changes of hair
2. Cataract
3. Changes of skin, intractable skin ulcers
4. Soft-tissue calcification
5. Bird-like face
6. Abnormal voice
Other signs and symptoms
1. Abnormal glucose and/or lipid metabolism
2. Deformation and abnormality of the bone
3. Malignant tumors
4. Parental consanguinity
5. Premature atherosclerosis
6. Hypogonadism
7. Short stature and low bodyweight

Cardiovascular and metabolic diseases including type 2 DM and lipid disorders have become prevalent in young patients. However, our patient was a non-obese male, without risk factors, who did not fit the expected clinical phenotype of these lifestyle disorders. Diagnosis of WS was suspected when in addition to metabolic diseases, the patient was noted to have some specific physical characteristics (history of cataracts, hair changes, bird-like face, and low body mass index). Genetic testing positive for WRN mutation completed the picture and helped confirm the diagnosis wherein the patient met all diagnostic criteria for WS. It is imperative to have a low threshold for considering progeroid syndromes in such atypical situations, wherein the initial presentation of atherosclerosis and multiple metabolic comorbidities generates a broad differential.

Classical WS is caused by homozygous or compound heterozygous loss of function mutations in the WRN gene [10]. More than 70 different disease mutations have been identified in classical WS patients from all over the world [11]. WRN protein is a part of RecQ family with both helicase and exonuclease activities and it participates in several cell metabolic pathways, including DNA repair and telomere maintenance [6,10]. WRN mutation status is determined using DNA-based techniques such as direct DNA sequencing and allele-specific polymerase chain reaction. A study conducted by Sadahira et al. found that immunohistochemical staining of erythroblasts could also be used as a rapid screening tool for detecting mutant WRN protein [12]. 

Medical advances may extend the life expectancy of patients with WS through better management of risk factors of atherosclerotic diseases and earlier diagnosis of malignant neoplasms [13].

## Conclusions

We believe that with further awareness regarding this disease and increasing availability of genetic testing new populations could be identified. This might potentially help identify novel founder mutations that can further add to our understanding and facilitate early recognition of this rare progeria syndrome. Our case will add to the literature about this rare condition and alsohighlights the importance of having a high index of suspicion for WS when initial clinical presentation is atypical.
